# Biological and Adhesive Properties of an Autologous Protein-Based Fibrin Sealant for Ophthalmological Applications

**DOI:** 10.1167/tvst.12.11.32

**Published:** 2023-11-28

**Authors:** Eduardo Anitua, Francisco Muruzabal, Roberto Prado, Ander Pino, Roberto Tierno, Mairobi Persinal-Medina, Mohammad H. Alkhraisat, Jesús Merayo-Lloves

**Affiliations:** 1 University Institute for Regenerative Medicine and Oral Implantology (UIRMI), Jacinto Quincoces 39, Vitoria, Spain; 2 BTI Biotechnology Institute, Jacinto Quincoces 39, Vitoria, Spain; 3 Instituto de Investigación Sanitaria del Principado de Asturias (ISPA), Avenida del Hospital Universitario, Oviedo, Spain; 4 Instituto Universitario Fernández-Vega, Fundación de Investigación Oftalmológica, Universidad de Oviedo, Avenida Doctores Fernández-Vega, Oviedo, Spain

**Keywords:** autologous fibrin sealant, plasma rich in growth factors, platelet-rich plasma, tissue regeneration, Tisseel

## Abstract

**Purpose:**

The aim of this study was to evaluate the biological and adhesive properties of a new autologous sealant based on plasma rich in growth factors (PRGF), named E-Sealant.

**Methods:**

Conventional PRGF and a commercial fibrin sealant (Tisseel) were included as controls. The hematological and protein content of E-Sealant was determined. Its bioactivity and biocompatibility were tested for human keratocytes (HKs). To evaluate its adhesion and regenerative capacity, E-Sealant was used on an animal model of conjunctival autograft surgery and compared to Tisseel.

**Results:**

E-Sealant presented a high growth factor content with levels similar to those of conventional PRGF. E-Sealant induced proliferative and migratory activity on HK cells equivalent to PRGF. Although autologous membranes induced the proliferation of HKs, cells cultured over Tisseel did not adhere nor proliferate. HK cells showed increased number and flattened morphology over PRGF and E-Sealant compared to scarce and round-shape cells detected in Tisseel. Conjunctival autograft glued with E-Sealant adhered successfully, whereas Tisseel application formed irregular clots. During follow-up, both adhesives showed good integration and no dehiscence. However, Tisseel-treated samples presented slightly increased hemorrhage and inflammation. In contrast to Tisseel, E-Sealant–treated autografts presented a continuous layer of non-keratinized stratified squamous epithelium. Inflammatory infiltrates were minimal in E-Sealant–treated conjunctiva, whereas the Tisseel group showed noticeable immune reactions. Unlike Tisseel-treated grafts, E-Sealant presented low immunoreactivity for smooth muscle actin (SMA), suggesting decreased fibrotic tissue formation.

**Conclusions:**

E-Sealant presents optimal biological and adhesive properties suitable for use as an ophthalmic glue, with regenerative purposes superior to commercial fibrin sealants.

**Translational Relevance:**

Our study analyzed the characterization and biological activity of a new autologous fibrin sealant in ocular surface cells and in an animal model in which the adhesive and regenerative properties of the fibrin sealant were evaluated.

## Introduction

The field of ophthalmology has witnessed remarkable progress over the last several years. Among the distinct innovative solutions that have revolutionized ophthalmic surgeries, tissue adhesives stand as an important advancement aimed at accelerating hemostasis and restoring ocular function.^1^ Injuries that do not undergo precise closing or that experience delayed wound healing after conventional suture may develop fibrotic tissue and inflammation; however, sealants provide ophthalmic surgeons with reliable tools for enhancing surgical outcomes and promoting faster healing. Hence, biologic glues have gained significant recognition due to their versatility and efficacy, positioning them as valuable alternatives to traditional closure methods.^2^

Bio-adhesives are biocompatible substances that adhere tissues together, promoting optimal sealing without the need for sutures or staples.^3^ In ophthalmology, tissue adhesives have found various applications, including corneal laceration repair, conjunctival closure, glaucoma surgery, retinal detachment surgery, or pterygium surgery.^4^^,^^5^ Currently, various commercially available adhesives are based on either natural or synthetic polymers.^6^ One of the most used synthetic sealants is cyanoacrylate glue. Cyanoacrylate polymerizes rapidly upon contact with moisture, creating a strong bond between tissue edges. This glue is particularly beneficial in the management of corneal perforations, where precise and quick wound closure is crucial to prevent complications and promote healing.^7^

Fibrin sealants are also widespread medical substances for ophthalmological applications. These adhesives, mainly derived from allogenic human plasma, mimic the final stage of the blood clotting process by combining concentrated fibrinogen, stabilized thrombin, and calcium ions. This mixture quickly forms a clot that effectively stops bleeding and creates a barrier that helps to seal the tissue.^8^ Fibrin glues have shown success in various ophthalmic procedures, including conjunctival closure, tissue graft fixation, and securing amniotic membranes during ocular surface reconstruction.^9^ However, like any medical intervention, synthetic adhesives and allogenic fibrin sealants present some limitations. Although minimal side effects have been reported, there is a risk of infection transmission and allergic response due to the presence of excipients and antifibrinolytic agents in their composition.^10^^,^^11^ Additionally, the economic cost of some commercially available adhesives is sometimes difficult to afford, given the limited shelf life and loss of efficacy over time.

For this reason, novel fibrin glues of autologous origin are attracting the attention of medical practitioners. The use of endogenous products obtained from the patient’s own blood has been shown to reduce adverse events and immune-mediated complications, offering a safer and a more biocompatible alternative.^12^ Moreover, as autologous sealants are usually related to platelet-derived bioproducts such as platelet-rich plasma (PRP), they present active substances within their composition rather than an inert fibrin scaffold.^1^ In fact, recent studies have demonstrated that the inclusion of platelet-derived growth factors and plasmatic proteins within autologous fibrin glues increases their final regenerative performance and promotes more efficient wound healing.^13^

Plasma rich in growth factors (PRGF–Endoret; BTI Biotechnology Institute, Vitoria, Spain) has proven its efficacy in the management of different ocular disorders and ophthalmological procedures.^14^ PRGF is a pure PRP (P-PRP), with a moderated enrichment in platelets, and is free of leucocytes; it is coded as 24-00-11 according to Kon et al.,^15^ and it is included in various classifications.^16^ In addition to the growth factor content, the absence of preservatives and stabilizers allows broad applicability in the ophthalmic field through the use of PRGF-derived formulations such as eye drops, injectables, fibrin clots, and transparent membranes.^17^ Recently, a new formulation was added to this ophthalmological armamentarium, which is subsequently referred to as “E-Sealant.” E-Sealant is an autologous fibrin glue that is obtained in situ based on PRGF technology.

In the present study, the biological properties of E-Sealant were characterized and analyzed in vitro on human keratocytes, and its adhesive and regenerative performance was tested in vivo on an animal model for conjunctival autograft attachment in pterygium surgery. Results have been compared against conventional PRGF formulations and one of the most widely used fibrin sealants on the market.

## Materials and Methods

### Obtaining the Different Fibrin Formulations

This study was conducted in accordance with the tenets of the Declaration of Helsinki. Blood donors were informed about this research and provided written informed consent. PRGF and E-Sealant were obtained from the blood of three male healthy donors*.* Briefly, blood was withdrawn and centrifuged at 580*g* for 8 minutes (System V; BTI Biotechnology Institute). The whole plasma column (PC) was then obtained, avoiding collection of the buffy coat. The concentrations of platelets and leukocytes were measured with a hematological analyzer (Pentra ES 60; Horiba ABX, Montpellier, France). The PC was then divided into four parts to obtain PRGF and E-Sealant: (1) one part of the PC was activated with calcium chloride (CaCl_2_; PRGF-Activator; BTI Biotechnology Institute) and incubated until clotting at 37°C to obtain a membrane (PRGF); and (2) the other three parts of the PC (PC1, PC2, and PC3) were used to obtain the E-Sealant as follows: PC1 was activated with CaCl_2_ and incubated at 37°C for a minimum of 40 minutes, allowing its coagulation and retraction, to obtain a supernatant (SP1). When it was necessary to use the E-Sealant in each assay, a mixture of CaCl_2_ and SP1 was added to PC2 favoring its coagulation after 2 to 3 minutes. After that, this mixture was shaken vigorously to generate a new supernatant (SP2). A ratio of 1 mL PC3 and 0.3 mL SP2 was then applied using a double syringe applicator (Nordson Medical, Westlake, OH) to obtain E-Sealant. The fibrin clot obtained from each formulation (PRGF and E-Sealant) was squeezed to obtain a final supernatant that was used to perform the growth factor and protein detection assays and the proliferation and migration assays. For the rest of the assays performed in the present study, the fibrin membrane obtained from each formulation was used. Plasma samples from different donors were not pooled.

In addition, Tisseel (Baxter Healthcare Corp., Deerfield, IL), one of the most widely used fibrin-based sealants on the market, was also used as a control for some of the assays carried out in the present work. Tisseel is a ready-to-use formulation and was used following the manufacturer’s instructions. In brief, the fibrinogen and thrombin constituents, precharged in a dual syringe device, were simultaneously injected using a dual syringe applicator, resulting in a dense formulation that gelled immediately after injection.

### Biological Properties

#### Growth Factor and Protein Measurement

Various growth factors and proteins related to tissue regeneration, including platelet-derived growth factor-AB (PDGF-AB), transforming growth factor β1 (TGF-β1), epithelial growth factor (EGF), and fibronectin (FN), were measured in the supernatants of both formulations (PRGF and E-Sealant) using commercially available colorimetric sandwich enzyme-linked immunosorbent assay (ELISA) kits (Quantikine ELISA Kits; R&D Systems, Minneapolis, MN). Absorbance changes were measured using a multimode microplate reader (Synergy H1; Agilent BioTek, Santa Clara, CA), and the concentrations of the different biomolecules were determined using Gen5 software (Agilent BioTek).

#### Cell Culture

Primary human keratocyte (HK) cells (ScienCell Research Laboratories, San Diego, CA) were cultured according to manufacturer's instructions as follow: Cells were grown to confluence in fibroblast medium supplemented with fibroblast growth supplement and fetal bovine serum (ScienCell Research Laboratories) in a humidified atmosphere at 37°C with 5% CO_2_. They were then detached using animal-free trypsin-like enzyme (Gibco TrypLE Select Enzyme; Thermo Fisher Scientific, Waltham, MA). The trypan blue dye exclusion test was used to analyze the cell viability.

#### Proliferation Assay

Keratocytes were seeded at a density of 8000 cells/cm^2^ on 96-well optical bottom black plates and were then cultured with supplement-free medium with 20% (v/v) PRGF or 20% (v/v) E-Sealant for 72 hours in triplicate. After that, the proliferation rates of HK cells treated with PRGF or E-Sealant were measured using the Invitrogen CyQUANT Cell Proliferation Assay Kit (Thermo Fisher Scientific). Briefly, the medium was removed from each well, and the cells were gently washed with PBS. Microplates were frozen at −80°C for at least 24 hours to ensure efficient cell lysis. The plates were thawed at room temperature before incubation with RNase A (1.35 Kunitz units [KU]/mL) and diluted in cell lysis buffer for 1 hour at room temperature. Subsequently, stain/cell lysis buffer (CyQUANT GR-dye) was added to each sample well, which was gently mixed and incubated for 5 minutes at room temperature without exposure to light. Fluorescence was measured using a multimode microplate reader (Synergy H1; Agilent BioTek).

#### Migration Assay

HK cells were seeded at high density in culture inserts (ibidi GmbH, Martinsried, Germany) to evaluate the potential of PRGF and E-Sealant to induce cell migration. The cells were placed on a 24-well plate and were then incubated until confluence. After that, the inserts were carefully removed, resulting in two cell monolayers separated by 500-µm thickness. Each well was then washed with PBS and incubated with PRGF or E-Sealant at 20% for 24 hours. Each treatment was applied in triplicate. Subsequently, the different treatments were removed, and the cell nuclei were stained by incubating them with Hoechst 33342 (Thermo Fisher Scientific) for 10 minutes. The central part of the septum before and after the treatment period was photographed in phase contrast and under fluorescence using a digital camera coupled to an inverted microscope (Leica DFC300 FX and Leica DM IRB; Leica Microsystems, Wetzlar, Germany) to quantify the number of migrating cells. ImageJ (National Institutes of Health, Bethesda, MD) was used to analyze the number of migrated cells found in the gap after 24 hours of treatment. The results were expressed in terms of the number of cells per square millimeter of the surface area.

#### Biocompatibility Assay

PRGF, E-Sealant, and Tisseel clots were obtained as described above and placed in the bottom of 96-well plates in triplicate. Samples were maintained at 37°C for 1 hour to ensure complete clotting. Then, HK cells were seeded onto the surface of the clots in complete culture medium at a density of 8000 cells/cm^2^ and maintained in a humidified atmosphere at 37°C with 5% CO_2_. Cell proliferation was evaluated using the WST colorimetric assay (Sigma-Aldrich) after 5 hours, which was considered as time point 0, when cell–substrate attachment occurs with no proliferation. The WST assay was subsequently carried out after 3, 7, and 10 days. At each time point, samples were incubated with WST reagent for 1 hour. Absorbance at 450/620 nm was directly proportional to the number of living cells. Clots without cells and cells cultured over standard wells without clots were used as controls. Results were expressed as a percentage of cell growth compared to the number of attached cells at time point 0 (100%).

In light of the results of the previous assay, additional samples after 3 days of treatment were preserved for scanning electron microscopy (SEM) analysis. Membranes were fixed with 2.5% glutaraldehyde, fixed with osmium tetroxide (1% OsO_4_ in 0.1-M cacodylate), and finally dehydrated through ascending alcohol concentrations. Thereafter, cell–clot constructs were subjected to critical-point drying (Autosamdri-814; Tousimis, Rockville, MD), gold sputter coated, and imaged using an electron microscope (S-4800; Hitachi, Tokyo, Japan).

### In Vivo Adhesion Study

#### Adhesive Formulations

The main objective of the in vivo study was to analyze the adhesive capacity and the biologic potential of the E-Sealant. These properties were compared to the gold-standard fibrin sealant Tisseel.

#### Animals

New Zealand White rabbits (males, 12 weeks old, 3.5–4.0 kg) were purchased from Granja San Bernardo (Navarra, Spain). All of the animals were treated according to the ARVO Statement for the Use of Animals in Ophthalmic and Vision Research. This study was approved by Oviedo University's Ethical Committee for Animal Studies and Asturias Animal Production and Health Service (Spanish registration code PROAE 36/2019 and PROAE 08/2020).

#### Surgical Procedure

To test the adhesion of E-Sealant versus Tisseel, conjunctival autograft surgery was used in an animal model. This model consists of removing the temporal conjunctiva and transplanting a free bulbar conjunctival graft to cover the exposed scleral tissue.^18^ A total of seven animals, four for the E-Sealant application (eight eyes) and three for Tisseel (six eyes), were used to evaluate the efficacy of the different adhesive formulations. All surgeries were performed under general anesthesia by the intravenous administration of 0.2 mg/kg medetomidine (Dormitor; Orion Corporation, Espoo, Finland) and 1 to 2 mg/kg diazepam (Valium 10; Hoffmann-La Roche, Basilea, Switzerland). In addition, 3% isoflurane was administered intranasally during the surgical procedure. Furthermore, 10 minutes before the procedure, double anesthesia of 0.1% tetracaine and 0.4% oxybuprocaine (Colircusi; Alcon Healthcare SA, Barcelona, Spain) was applied. After that, a vasoconstrictor solution of 1 mg/mL adrenaline (B. Braun Medical, Barcelona, Spain) was injected under the temporal conjunctiva to separate the conjunctiva and underlying Tenon's capsule from the sclera, and it was then excised using Wescott scissors, exposing a bare 4 × 5-mm scleral bed. Subsequently, an area of 5 × 6 mm was marked from the superior bulbar conjunctiva to obtain a free autograft. The epithelial side and the juxtalimbar border were also marked to prevent graft inversion and disorientation. To balloon out the conjunctival autograft area and separate it from the underlying Tenon's capsule, a vasoconstrictive solution (0.1 mL) was injected under the donor conjunctiva. The conjunctiva was then carefully dissected, with special care taken to avoid buttonholes and graft rollover. After that, 0.2 mL of E-Sealant or Tisseel was applied on the bare sclera, and the conjunctival autograft was immediately transferred onto the bare sclera. An additional 0.2 mL of E-Sealant or Tisseel was applied to the edges of the graft bordering the healthy conjunctiva and cornea. Passive blinking was induced to verify graft attachment after a coagulation period of 5 minutes. Finally, to prevent ocular damage due to animal behavior, tarsorrhaphy was performed with a 4-0 nylon suture. Three days after surgery, the sutures were removed, and the surface of the eye was washed with a balanced salt solution (BSS; Medical Mix SLU, Barcelona, Spain).

During the 7 days following the procedure, daily subcutaneous administration of antibiotic (5–10 mg/kg enrofloxacin; Ecuphar Veterinaria, Barcelona, Spain) and analgesic (0.3 mg/kg meloxicam, METACAM; Boehringer Ingelheim, Barcelona, Spain; 0.01–0.05 mg/kg buprenorphine; Bupaq, Richter Pharma AG, Wels, Austria) was applied to each animal. In addition, topical eye drops containing an antibiotic (0.3% tobramycin; Tobrex, Alcon Healthcare SA) and a glucocorticoid (0.1% dexamethasone, Maxidex; Alcon Healthcare SA) were applied three times a day from day 3 to day 14.

#### Clinical Follow-Up

Clinical follow-up of all rabbits was carried out from the opening of the tarsorrhaphy (day 3) until day 14. Clinical evaluation consisted of assessment of the presence of residual adhesive formulation (E-Sealant or Tisseel), dehiscence, inflammation, hemorrhage, retraction, integration, and vascularization. At the end of follow-up, all rabbits were sedated with medetomidine and diazepam (as described above) and euthanized by intravenous pentobarbital sodium overdose. Finally, the eyes were enucleated and fixed in 4% paraformaldehyde for 24 hours for histological evaluation.

#### Histological Analysis

The temporal conjunctiva area in which the autograft was placed was dissected from enucleated eyes and embedded in paraffin. Afterwards, 6- µm sections were cut from the blocks, and the different samples were stained with hematoxylin and eosin (Sigma-Aldrich) and Masson's trichome (Bio-Optica, Milano, Italy). Several photographs were captured with different augmentations with a digital camera coupled to a Leica DM IRB optical microscope (Leica Microsystems). In addition, immunohistochemistry for the detection of alpha-smooth muscle actin (α-SMA) was performed to analyze the presence of myofibroblasts in conjunctival autografts treated with E-Sealant or Tisseel. For this purpose, the sections were deparaffinized, rehydrated, and rinsed with PBS. Endogenous peroxidase activity was then blocked with 3% hydrogen peroxide for 10 minutes, and the slides were washed twice with PBS for 5 minutes each time. A background blocking was performed with 10% fetal bovine serum for 30 minutes, and then the sections were incubated with the mouse primary antibody for SMA (Abcam, Cambridge, UK) diluted 1:800 in PBS for 1 hour. The samples were then washed twice with PBS for 5 minutes each time and incubated with a ready-to-use biotinylated anti-mouse secondary antibody (Abcam) for 30 minutes. The sections were then washed twice with PBS for 5 minutes and incubated for 30 minutes with a ready-to-use peroxidase streptavidin (Abcam). Samples were washed with PBS, and peroxidase activity was revealed using the Vector VIP kit (Vector Laboratories, Newark, CA). Finally, sections were counterstained with hematoxylin, mounted, and visualized and photographed using a Leica DMLB microscope equipped with a Leica DFC310 FX digital camera.

### Statistical Analysis

Results are expressed as mean ± SD. After testing data normality (Kolmogorov–Smirnov and Shapiro–Wilk). Depending on the number of treatment groups used in each assay, different comparison tests were carried out to evaluate the differences between the groups. In the case of the assays performed with two treatment groups, the Student's *t*-test or the non-parametric Mann–Whitney test was applied, depending on the normality test results. For assays carried out with more than two treatments groups, possible differences among the treatment groups were analyzed using ANOVA tests of variance and a subsequent post hoc analysis for multiple comparisons among groups. In cases where no normality was observed, the nonparametric Kruskal–Wallis test with a subsequent Mann–Whitney analysis test for multiple comparisons between groups were used. *P* < 0.05 was considered the level of statistical significance. All statistical analyses were performed with the SPSS Statistics 15.0 (IBM, Chicago, IL).

## Results

### Biological Characterization

The whole PC from which PRGF and E-Sealant were obtained had a platelet concentration of 369 ± 115 × 10^3^ platelets/µL and had almost no leukocytes (0.1 ± 0.2 × 10^3^ leukocytes/µL). Platelet enrichment was 1.5- ± 0.3-fold when comparing the platelet content of the PC with that of peripheral blood. The results of protein and growth factor concentrations of PRGF and E-Sealant are presented in the Table. In summary, no significant differences (*P* > 0.05) were observed between PRGF and E-Sealant regarding the different proteins and growth factors related to tissue regeneration.

**Table.** Concentrations of Growth Factors and Proteins Evaluated in the Various Formulations

	Mean ± SD			
	EGF (pg/mL)	PDGF-AB (pg/mL)	TGF-β1 (pg/mL)	FN (ng/mL)
PRGF	409 ± 80	10404 ± 3773	20967 ± 5853	147 ± 66
E-Sealant	434 ± 88	10805 ± 4153	21467 ± 4272	180 ± 59

EGF, epithelial growth factor; PDGF-AB, platelet-derived growth factor-AB; TGF-β1, transforming growth factor-β1; FN, fibronectin.

### Biological Activity

**Figure 1.**
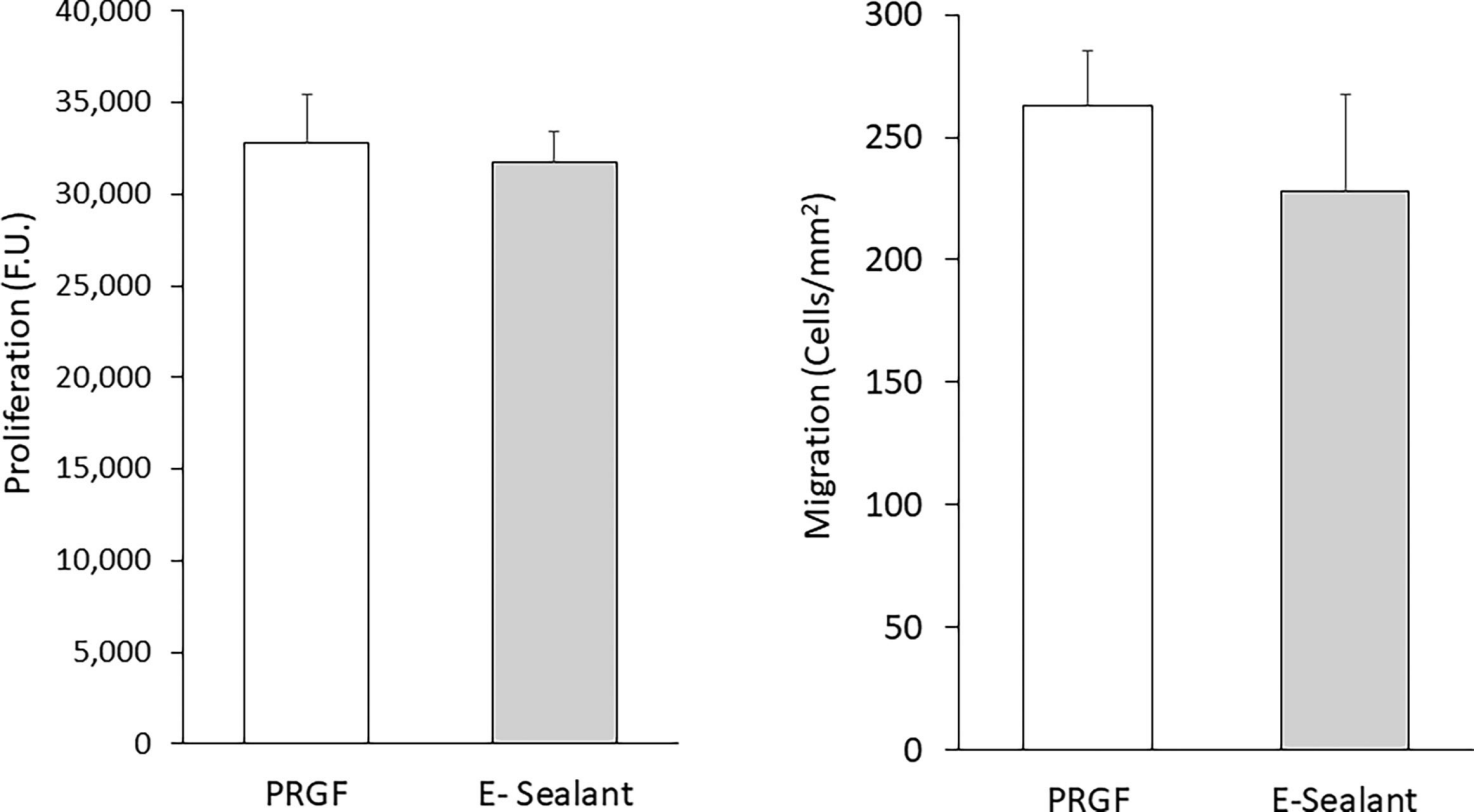
Biological activity of HKs after culture with the supernatant obtained from PRGF or E-Sealant. No significant differences were observed either in proliferative activity, expressed as arbitrary fluorescent units (FU), or in migration, expressed as number of cells per square millimeter. Data are reported as mean ± SD.

The biological potential of PRGF and E-Sealant was analyzed over HK cell proliferation and migration (Fig. 1). The proliferation assay showed that both formulations induced similar proliferative activity in HK cells (*P* > 0.05). On the other hand, although the migration index was slightly lower in HK cells treated with E-Sealant with respect to PRGF treatment, no statistically significant differences were observed between the two formulations.

### Biocompatibility Assays

**Figure 2.**
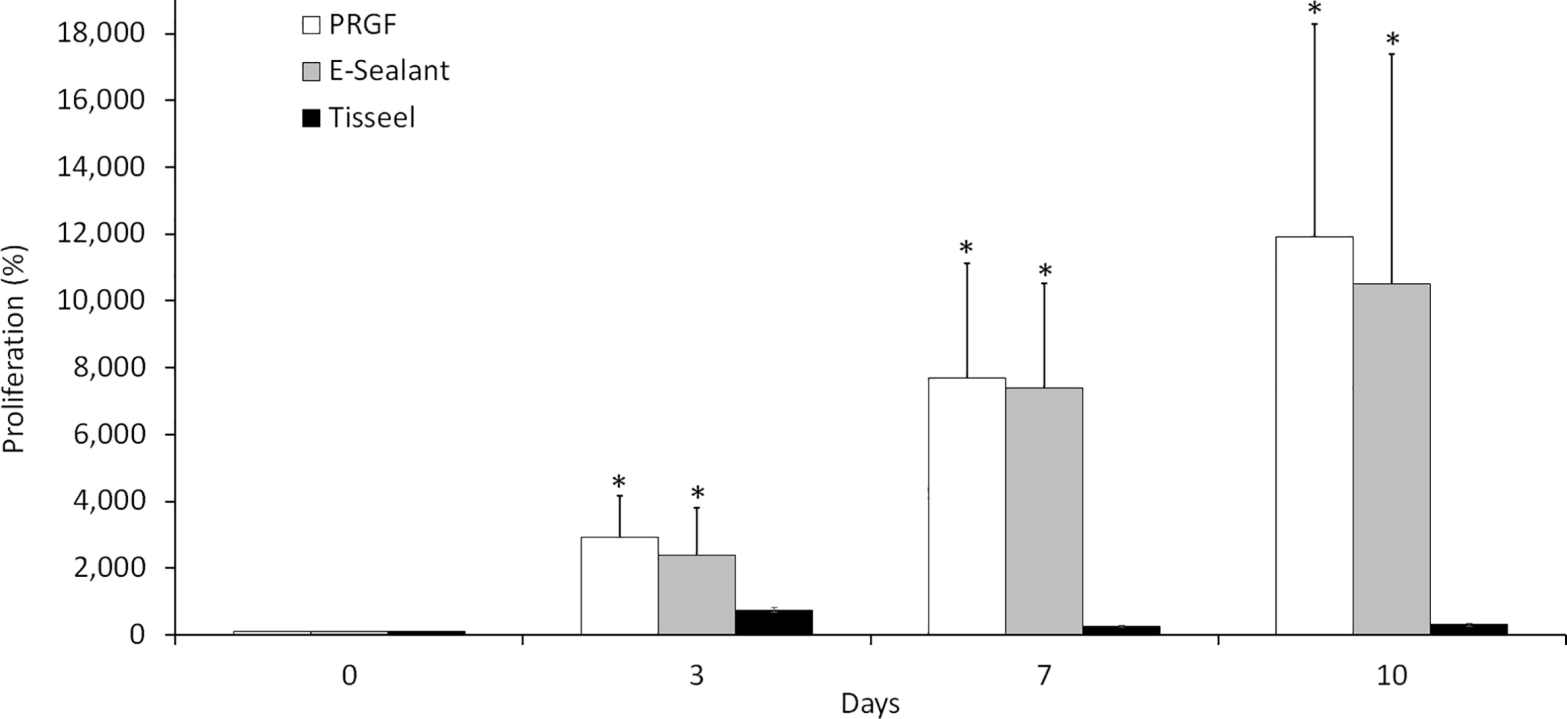
Biocompatibility assay of HKs cultured onto the surface of clots obtained from PRGF, E-Sealant, and Tisseel. Cell proliferation was analyzed at 0, 3, 7, and 10 days. Data are expressed as mean ± SD. *P < 0.05 with respect to Tisseel.

HK cells were seeded onto the surface of clots obtained from PRGF, E-Sealant, and Tisseel formulations and analyzed for cell proliferation at 0, 3, 7, and 10 days. The proliferation assay showed that the number of HK cells increased along each time point after treatment with PRGF or E-Sealant (Fig. 2). Tisseel induced an increase in the number of HK cells after 3 days of treatment; however, the number of cells growing onto Tisseel decreased at the following time points (7 and 10 days). Statistically significant differences were observed in the number of HK cells after treatment with PRGF and E-Sealant compared to Tisseel at each study time point (3, 7, and 10 days). No significant differences were observed in the proliferation rates of HK cells seeded onto PRGF or E-Sealant membranes throughout the study period (Fig. 2).

**Figure 3.**
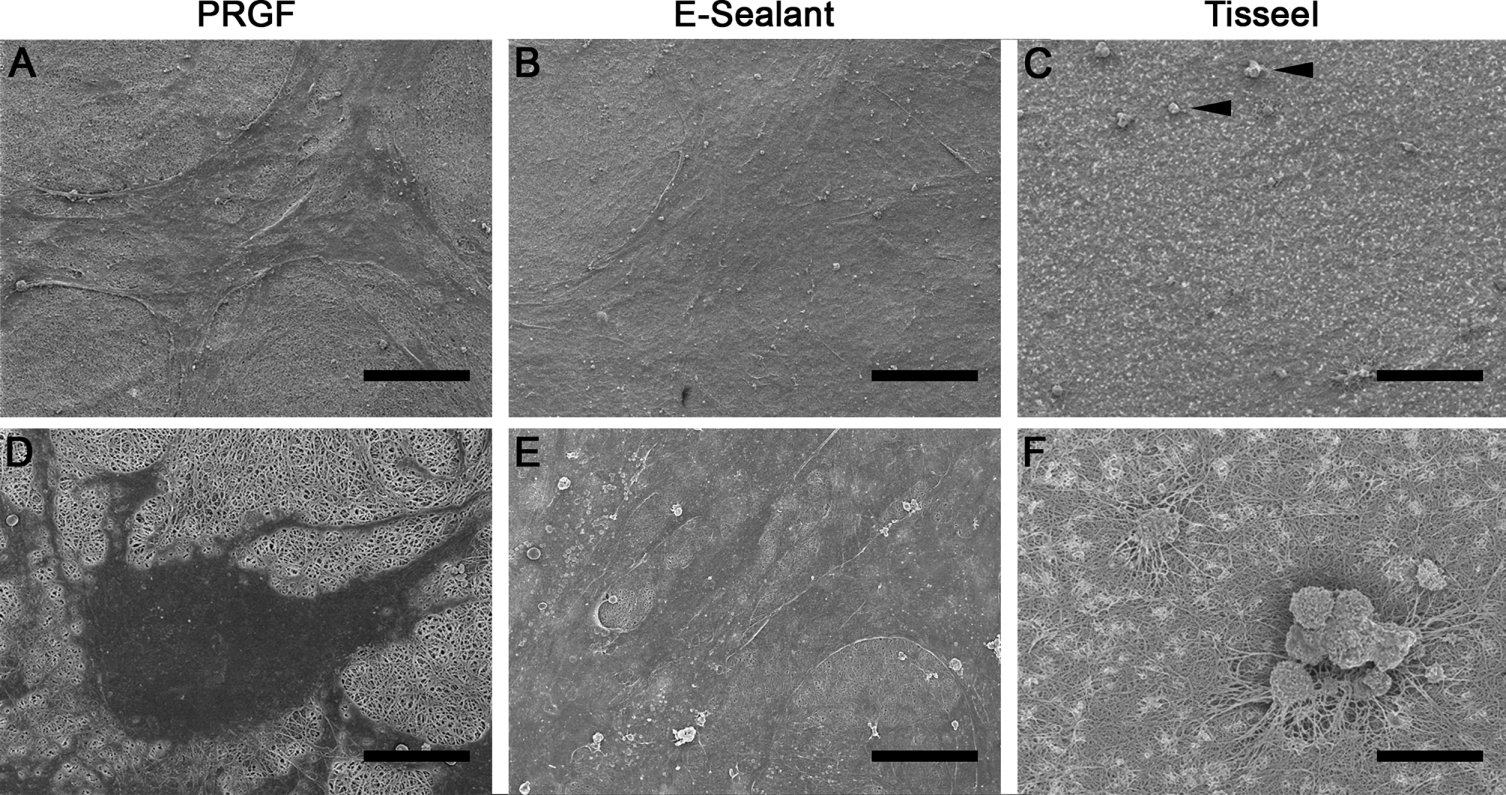
SEM images of HK cells cultured over the fibrin membranes for 3 days. The cells grew onto the surface of clots obtained from PRGF, E-Sealant, and Tisseel. (**A**–**C**) Distribution of cells at low magnification. (**D**–**F**) Details of the morphology of cells cultured on these membranes at high magnification. The *arrowhead* in panel C shows the rounded cells on the surface of Tisseel, in contrast to the elongated and fully adherent cells in the PRGF and E-Sealant formulations. Original magnifications: 250× (**A**–**C**); 1200× (**D**–**F**). *Scale bars*: 100 µm (**A**–**C**); 20 µm (**D**–**F**).

Figure 3 shows representative SEM images of HK cells cultured over the fibrin membranes obtained with the different formulations (PRGF, E-Sealant, and Tisseel) for 3 days. The images reveal a higher number of HK cells in the samples cultured on the matrices obtained from PRGF and E-Sealant compared to the samples obtained from Tisseel (Figs. 3A–3C). These results are in accordance with those obtained in the proliferation assay. In addition, HK cells cultured on PRGF and E-Sealant membranes adopted a flattened morphology with several cytoplasm elongations that would allow for increased cell adhesion to the fibrin membrane (Figs. 3D, 3E). However, the morphology of HK cells cultured on Tisseel had a totally different morphology, adopting a rounded shape (Fig. 3F).

### In Vivo Adhesion Study

#### Intraoperative and Clinical Follow-Up

The application of both fibrin sealants (E-Sealant and Tisseel) during the surgical period allowed high extrusion control and placement over the bare sclera and the junction points between the graft and the surrounding conjunctiva. E-Sealant produced a firm, sticky clot within 1 to 2 minutes of application, sufficient time to allow the clinician to place the conjunctival autograft before complete coagulation occurred. Conjunctival autograft glued with E-Sealant adhered successfully and demonstrated sufficient adhesion strength to maintain graft integrity without folding or dehiscence during postoperative passive blinking. On the other hand, although the Tisseel application produced a firm graft adhesion, its coagulation was instantaneous after its application and the working time was insufficient, with irregular clots forming.

**Figure 4.**
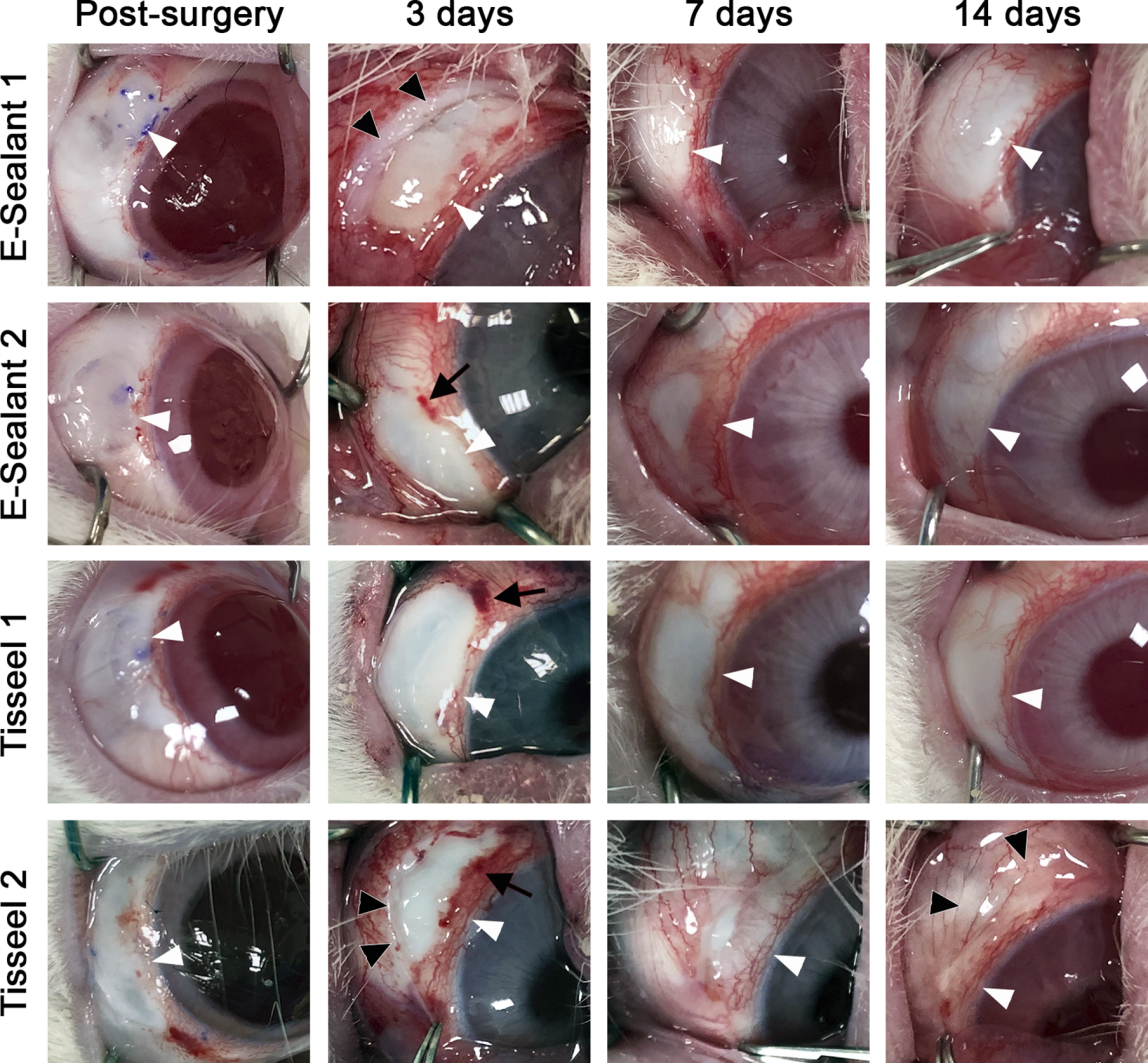
Image gallery showing the evolution of rabbit eyes in two representative cases treated with E-Sealant and two others treated with Tisseel. The eyes are shown immediately after surgery and after 3, 7, and 14 days. *White arrowheads* indicate the localization of the autologous conjunctival grafts, *black arrows* show the presence of hemorrhage, and *black arrowheads* indicate inflammation of the surrounding conjunctiva.

Three days after surgery, all conjunctival autografts in both treatment groups were perfectly integrated within the surrounding tissue, and none of them showed graft dehiscence from the scleral bed (Fig. 4). Subconjunctival hemorrhage around the graft was observed in 35% and 50% of grafts treated with E-Sealant and Tisseel, respectively; however, it resolved spontaneously without complications in the following days. In addition, 20% of grafts treated with E-Sealant and 50% of those treated with Tisseel showed inflammation of the conjunctival tissue surrounding the graft, which was resolved in the next follow-up time evaluation. At final follow-up (14 days), all autografts appeared vascularized and integrated within surrounding conjunctival tissue without adverse corneal or conjunctival effects. On the other hand, some Tisseel-treated grafts showed mild to moderate signs of inflammation in the graft and surrounding conjunctiva, with one graft showing significant inflammation (Fig. 4). In contrast, no significant inflammation was observed in the E-Sealant–treated rabbit eyes. In addition, some eyes treated with Tisseel presented a clear conjunctival hyperemia that was already present at 3 days around the autograft and increased after 7 and 14 days, during which hyperthermia was also observed in the autograft.

#### Histological Analysis

**Figure 5.**
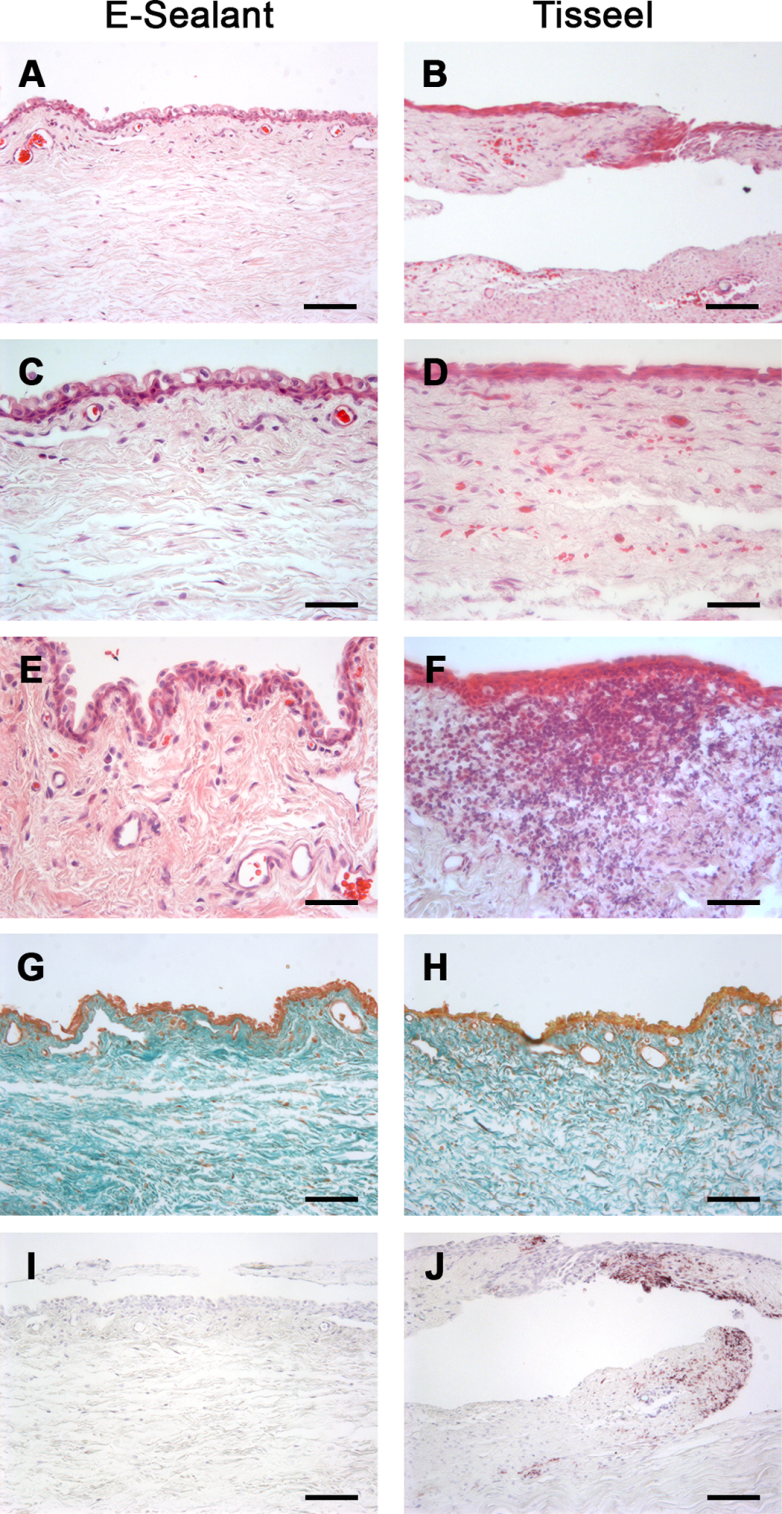
Histological analysis of rabbit corneas treated with E-Sealant (*left column*) and Tisseel (*right column*) after 14 days of follow-up. (**A–F**) Samples of conjunctival autografts stained with hematoxylin and eosin. (**G**, **H**) Masson's trichome microphotographs showing the distribution of collagen fibers in the conjunctiva. (**I**, **J**) Immunohistochemistry for α-SMA protein demonstrating the presence of myofibroblasts in the Tisseel-treated conjunctival autografts in contrast to the samples treated with E-Sealant, where no staining for SMA was observed. *Scale bars*: 50 µm (**A**, **B**, **G–J**); 100 µm (**C**–**F**).

Histological analysis of the samples stained with hematoxylin and eosin showed that all E-Sealant–treated conjunctival autografts were well integrated into the bulbar conjunctiva and also revealed a continuous layer of non-keratinized stratified squamous epithelium (Figs. 5A, 5C). However, some discontinued epithelia were observed in some samples of the autograft treated with Tisseel (Fig. 5B). In addition, inflammatory infiltrates were absent or minimally present in the conjunctiva of E-Sealant–treated grafts (Fig. 5E), whereas the Tisseel group showed mild to severe immune reactions (Fig. 5F).

Masson's trichrome showed no differences between the conjunctival autograft treated with either fibrin sealant (E-Sealant or Tisseel), and there were similar distributions of collagen fibers in the conjunctiva of all samples (Figs. 5G, 5H). The presence of myofibroblasts in the grafts was evaluated by immunohistochemical detection of SMA. The persistence of myofibroblasts in the repaired tissue may lead to the development of scars that would hinder tissue functionality. Immunoreactivity for SMA was minimal or absent in E-Sealant–treated conjunctival autografts (Fig. 5I), whereas some Tisseel-treated samples showed a high immunoreactivity for SMA, suggesting initial fibrotic tissue formation in the conjunctival graft treated with Tisseel (Fig. 5J).

## Discussion

Surgical procedures or accidents can cause tissue injury and require innovative efforts to accelerate hemostasis and wound healing. Today, the gold standard for wound closure is the use of sutures, staples, clips, or strips.^19^ However, none of these treatments is without certain drawbacks, including being time-consuming procedures and the risk of stitch inflammation. Scar formation, pain development during their removal, and their limited use depending on the severity of the wound are additional limitations.^1^ In the last several decades, various tissue adhesives have been developed to address these problems and the unmet medical needs generated by the exponential increase in the number of surgeries performed each year. These tissue adhesives must achieve certain characteristics, such as mechanical strength and adhesive properties, but they should also be biocompatible, sterile, biodegradable, effective for wound healing, easy to prepare, easy to use, and cost effective.^20^^,^^21^

Today, various tissue adhesives are widely used in different medical fields. They are applied in ophthalmology for the treatment of different ocular pathologies and as adjuvants in several surgical procedures such as corneal laceration repair, conjunctival closure, glaucoma surgery, retinal detachment surgery, or pterygium surgery.^4^ Due to their high biocompatibility compared to other sealants, fibrin-based adhesives are one of the most widely used tissue glues in ophthalmology. Most of them have an allogeneic origin and are based on a pool of plasma obtained from several donors that enriches and isolates the main constituents of the fibrin sealant (fibrinogen and thrombin) from the rest of the plasma substances (proteins, growth factors, and lipids, among others).^22^ The main disadvantage of this type of product is precisely its allogeneic origin, which increases the risk of transferring infections by bloodborne pathogens.^23^ To avoid this problem, Fernández-Vega-Cueto et al.^24^ recently described a process to obtain an autologous fibrin sealant. It was obtained from a PRP; however, as in the case of most fibrin sealants of allogeneic origin, fibrinogen and thrombin were isolated from the rest of the plasma components. Consequently, proteins and growth factors found within its secretome which are involved in tissue regeneration are absent. As a result, the biological activity and biocompatibility of this type of tissue adhesive may be reduced.

In the present study, the biological characterization, biological activity, and adhesion of a new autologous fibrin sealant (E-Sealant) obtained by a simple protocol using PRGF technology was evaluated in comparison with the usual PRGF fibrin membrane and Tisseel. This new autologous fibrin sealant was obtained from the whole plasma column of the PRGF and its supernatant. PRGF is a type of PRP with a moderate concentration of platelets, ranging from 1.5- to 2-fold over the peripheral blood. After platelet activation, myriad proteins and growth factors involved in the various processes of tissue regeneration are released to the PRGF supernatant.^25^^,^^26^ The present study found no differences in protein and growth factor concentrations between supernatants obtained from the conventional PRGF membrane and the PRGF sealant (E-Sealant), suggesting that the proteins and growth factors contained in the PRGF formulation are not altered, either qualitatively or quantitatively, during the process of obtaining E-Sealant.

Several studies have shown that PRGF increases the biological activity of different cell types obtained from distinct tissues, including cells from ocular surface tissues, such as HK cells.^27^^,^^28^ The results obtained in this work indicate that E-Sealant stimulates HK cell proliferation and migration at levels comparable to those of PRGF, suggesting that E-Sealant preserves the biological activity attributed to PRGF.

Here, analysis of growth factors and proteins and the proliferation and migration assay could not be performed with the supernatant obtained from the Tisseel fibrin membrane because the Tisseel formulation lacks proteins and growth factors related to tissue regeneration.^29^^,^^30^ Instead, a biocompatibility assay was performed by seeding HK cells on the fibrin membranes obtained from the different formulations (PRGF, E-Sealant, and Tisseel) and culturing them for 3, 7, and 10 days. The results obtained in this assay showed that E-Sealant and PRGF increased the number of HK cells at each time point of the study; the number of HK cells cultured on Tisseel increased slightly at 3 days after the start of treatment, but their number decreased steadily in the following days. SEM analysis of HK cells cultured on the different fibrin membranes after 3 days of treatment showed a high number of HK cells on the PRGF and E-Sealant fibrin membranes, which adopted a flat shape with multiple elongations of the cytoplasm, suggesting adequate cell adhesion to the fibrin membrane. However, the number of HK cells cultured on Tisseel membrane was considerably lower, and the cells adopted a rounded morphology that would be in line with lower adhesion to the fibrin membrane. These results are consistent with previous studies demonstrating that a high concentration of fibrinogen and thrombin inhibits the proliferation and spreading of cells, which adopt a rounded shape as in the case of Tisseel.^31^^–^^33^

Clinical follow-up demonstrated reasonable ocular tolerability with no corneal or conjunctival adverse effects when using the E-Sealant formulation. In contrast, some of the conjunctival autografts treated with Tisseel showed a greater degree of inflammation in the surrounding conjunctiva. The histologic study confirmed that Tisseel induced a greater infiltration of inflammatory cells compared to E-Sealant. These results are in accordance with those recently found by Fernández-Vega-Cueto et al.,^24^ who observed that Tisseel induced a greater infiltration of immunogenic components than autologous fibrin, attributing these results to a greater xenogeneic response due to the higher concentration of fibrinogen in Tisseel.^24^ On the other hand, E-Sealant–treated specimens showed normal conjunctival and epithelium with minimal presence of immune cells, no edema, and no scarring. However, Tisseel-treated samples showed areas where complete re-epithelialization and adhesion of the graft to the recipient stroma had not occurred. They also showed increased expression of SMA and myofibroblasts, which could lead to scarring and reduced functionality of the affected tissue. These results may be related to the presence of proteins and growth factors in E-Sealant which would accelerate wound healing while reducing scar formation.^34^

This study has some limitations related to the low number of blood donors used and the low number of eyes employed in the experimental model. In addition, the application of fibrin sealants of human origin could induce an exacerbation of the inflammation process because of xenogeneic reactions. However, the results observed in the present study suggest that E-Sealant has biological activity similar to that of PRGF, mainly due to the presence of growth factors and proteins involved in tissue regeneration. Moreover, it is important to highlight that the main advantage of E-Sealant over PRGF is that E-Sealant significantly reduces clotting time and increases the tissue adhesion properties of fibrin. In addition, E-Sealant favors the adhesion and proliferation of HK cells and the adhesion of conjunctival autograft in comparison to Tisseel, accelerating the wound healing while reducing the scar formation. Finally, E-Sealant presents optimal biological and adhesive properties suitable for its use as an ophthalmic glue, with regenerative properties superior to commercial fibrin sealants.
